# Ethical Issues Surrounding Newborn Screening

**DOI:** 10.3390/ijns7010003

**Published:** 2021-01-09

**Authors:** R. Rodney Howell

**Affiliations:** Department of Pediatrics, The Hussman Institute for Human Genomics and Genetics, Miller School of Medicine, University of Miami, Coral Gables, FL 33146, USA; rhowell@miami.edu

**Keywords:** parental advocacy history, residual dried blood spots, newborn screening expansion, recommended uniform screening panel (RUSP), secretary’s advisory committee of heritable disorders in newborns and children (ACHDNC)

## Abstract

It would be difficult to overestimate the importance of persistent, thoughtful parents and their importance in the development of treatments for their children’s rare disorders. Almost a century ago in Norway, observant parents led a brilliant young physician-scientist to his discovery of the underlying cause of their children’s profound developmental delay—i.e., phenylketonuria, or PKU. Decades later, in a recovering war-ravaged Britain, an equally persistent mother pressed the scientists at Birmingham Children’s Hospital to find a way to treat her seriously damaged daughter, Sheila, who suffered from PKU. Living on the financial edge, this mother insisted that Bickel and colleagues develop such a diet, and she volunteered Sheila to be the patient in the trial. The scientists concluded that the low phenylalanine diet helped but needed to be started very early—so, newborn screening was born to permit the implementation of this. Many steps brought us to where we are today, but these courageous parents made it all begin.

Although many intricacies surround the ethical issues in newborn screening, it is interesting from my perspective to have seen the evolution from no discussion of ethical issues to many discussions taking place in 2020 and consider the basis of this change.

It has been a century since an observant young mother in Norway, Borgny Egeland, and her dentist husband, Harry, were very concerned about the developmental delay of their 3-year-old daughter. Soon, a son was born who showed similar severe delay. They recognized a strange odor, and felt this odor represented a chemical abnormality in these children. Ms. Egeland sought out a professor who had taught Harry in dental school, the physician Asbjørn Følling, who had an interest in ketosis. Strongly aided by Ms. Egeland’s extensive urine collection, Følling was able to isolate an unusual compound which was responsible for the striking evanescent green color with ferric chloride, a compound routinely used to detect ketones in urine. He laboriously but rapidly determined that the unusual compound was phenylpyruvic acid, and he determined that the children had very high concentrations of phenylalanine, an essential amino acid, in the blood. A quick survey of institutions housing developmentally delayed children by Følling determined that a portion of profoundly delayed children had similar chemical abnormalities, and it appeared to be inherited in an autosomal recessive fashion. Enormous excitement surrounded the discovery of a chemical abnormality which caused this profound delay. It was immediately considered that if the elevated phenylalanine was causing the delay, removing this essential amino acid should be beneficial.

Følling’s remarkable discovery was published in 1934 [1], and in the ensuing years various attempts were made to modify the diet but nothing organized or effective was soon recognized. The Second World War was moving rapidly across Europe so biomedical research was greatly slowed. Much later, in the 1950s, Horst Bickel, a German Physician Scientist working in England, carried out a remarkable effort to treat a young child with classic phenylketonuria with a modified casein hydrolysate diet rendered low in phenylalanine [2]. Fortunately, Louis Woolf, working with a London drug house on special diets for war survivors, provided information to Bickel as to how to prepare such a diet (Dr. Woolf, retired in British Columbia, Canada, has recently celebrated his 100th birthday and has been widely recognized for his contributions in this area) [3]. Although the Bickel study showed strong improvement of the treated young child, it was thought that one would have to begin very early in order to get the best results.

The need to begin the lower phenylalanine diet very early brought Robert Guthrie on board, and he developed a simple but effective screening test, suitable for testing the entire population of newborns to identify babies with elevated phenylalanine—and so, newborn screening was born. Although there were many things to be learned, the identification and treatment of apparently healthy newborn infants with dramatic elevations of blood phenylalanine concentrations in the newborn period was clearly effective. The excitement was widespread and newborn screening was rather quickly begun across the entire United States. The ability to prevent severe developmental delay with a special diet was remarkable, and certainly at that time, there were no substantial voices opposing this remarkable new effort. This dramatic discovery resulted in national excitement with widespread publication. Our President of the United States at the time, President John F. Kennedy, keenly interested and involved in developmental delay because of his sister Rosemary, invited Asbjørn Følling to the White House where he was presented with a Joseph P. Kennedy, Jr. Foundation Award. Of course, there was the rare voice that cautioned that infants would be harmed or even killed. I was personally involved in treating children with this special diet very early in newborn screening. We would monitor blood phenylalanine concentrations carefully, and at times would find treated children slowed in growth, and/or who developed anemia, and we would increase phenylalanine in the diet and reverse this problem. It was miraculous to see children with essentially normal abilities (and progressively getting better as we learned more about the diet) when we had untreated children we were seeing in our clinic with profound developmental delays. A comprehensive review by medical historians found little evidence of death or disability that resulted from the inappropriate treatment of well children who were falsely identified by early newborn screening programs [4].

The dramatic effects in treating phenylketonuria (PKU) was not accompanied with any substantial discussion of ethical issues surrounding newborn screening. Over the ensuing 50 years, newborn screening has expanded greatly. The Secretary’s Advisory Committee of Heritable Disorders in Newborns and Children (SACHDNC) was formed by federal legislation and signed into law by President Bush in 2007 and requires (using evidence-based guidelines) what should be on the recommended uniform panel across all states. This Committee’s recommendations have extensively changed the face of inborn errors of metabolism as well as other conditions detected in the newborn period [5]. The establishment of the Recommended Uniform Panel and its adoption widely has, unfortunately, developed programs with low positive predictive values in some states, meaning that there was a dramatic increase in false-positive newborn screening results. While dealing with the many false-positive results of complex disorders, there has been increased interest in informed consent for all of newborn screening. At the same time, information provided to parents about newborn screening has been variable and provided at times of great stress during delivery of a baby, and so is usually not retained.

Although the residual dried blood spots (DBSs) from newborn screening have long been retained in many (most) states for laboratory quality control and for establishing new tests recommended by federal and state advisory panels, this has become a most contentious issue. I think that this derives from two reasons: the families were not advised that these spots were being retained for the purposes mentioned, and a misunderstanding of what can be achieved with such spots. Another major issue has developed when some became aware that the dried blood spots contained DNA, which caused serious concern among some. Protestors, I think, felt that a laboratory can identify a person from the DNA in the dried spot, which for practical purposes cannot be performed, and would serve no purpose if it could [6].

This author does not think that any competent parent, who understands inheritance would ever deny newborn screening for their infant. A common comment that screening is denied because nobody in the family has a genetic disease shows a serious lack of understanding. I do think that if the residual dried blood spots are to be used for identifiable research, the parents must be asked for permission to do so. I also think that very hard work to provide the best possible information to parents about newborn screening will go a long way to help parents understand the process, and feel comfortable that the best is being offered for their infant. The conditions that are being studied to become a part of the recommended uniform screening panel should be carefully studied with the best information available ensuring that the condition has a reliable test and an effective treatment. It is also important to understand the clinical course of a disorder, but at the same time be fully aware that one never knows the spectrum of any human disorder until one carries out screening on the apparently healthy unselected population. We have regularly seen the dramatic changes in our understanding of a disease once we have begun population-based newborn screening.

How informed consent is employed in newborn screening has been written about since the very earliest days of newborn screening in the United States [7,8]. The work of Faden cited here is widely discussed in the Bush-Bodamer manuscript in this Special Issue. My personal attitude about newborn screening is that there should be excellent material presented to all parents (and guardians) in an effort to convey understanding about the process overall. In this environment, I do not think that newborn screening for conditions recommended by the ACHDNC should require informed consent. Any condition for which screening is performed which does not meet these criteria I think should require full informed consent.

The widespread availability of whole-genome and whole-exon sequencing in clinical practice and the continuing significant decrease in cost of sequencing have raised the question of whether traditional metabolic newborn screening might be replaced by genome sequencing [9]. It has been clearly shown that DNA amplified from both old and new dried blood spots performs as well as their whole-blood reference samples with regard to error rates, indicating that DBSs are excellent sources of DNA for next-generation sequencing studies of disease [10]. Such studies have led to the widespread use of DBSs for second-tier confirmatory targeted next-generation sequencing.

Recent outstanding therapies for Duchenne Muscular Dystrophy (DMD) will likely result in this condition being nominated in the near future for inclusion on the Recommended Uniform Panel for newborn screening in the United States [9]. Two pilot newborn screening programs for this condition are already the subject of state-wide research programs. Interestingly, the current FDA approved drugs for DMD are mutation specific, meaning that the specific mutation must be known before treatment can begin. Therefore, whole-genome sequencing should logically follow when an infant has an initially positive newborn screening test. This could be the first major use of widespread sequencing, albeit secondary, in the NBS lab.

There is an enormous difference between the falling cost of genome sequencing and of mass spectroscopy. This will be an enormous impediment in the foreseeable future as far as using whole-genome and whole-exon sequencing as the primary newborn screening tools, but this may change rather quickly.

A most difficult situation will be the enormous issues with obtaining fully informed consent for broad sequencing of mostly healthy babies. Analyzing the genomic information of millions of healthy babies will present an enormous technical, as well as ethical, challenge for the foreseeable future. Recent studies have addressed some of the issues that arise when whole-exome/whole-genome sequencing is included in the newborn screening program. These authors point out that availability of genomic data require families to make decisions about information that may predict future events, with differing levels of certainly, ability to intervene, and may involve conditions that may not allow direct interventions among other challenges [11]. A diverse group of experts were brought together to consider these many challenges to the introduction of whole-genome sequencing into newborn screening [12,13]. We have not yet faced such an enormous challenge at a population level.

## References

[1] Følling A. (1934). Excretion of phenylpyruvic acid in urine as a metabolic anomaly in connection with imbecility. Nord. Med. Tidskr..

[2] Bickel H., Gerrard J., Hickmann E.M. (1954). The influence of Phenylalanine Intake on the Chemistry and Behaviour of Phenylketonuric Child. Acta Paediatr..

[3] Howell R.R., Sinclair G. (2020). A Visit with Dr. Louis Woolf, Recognizing his 100th Birthday and His Contributions to the Diagnosis and Treatment of Phenylketonuria. Int. J. Neonatal Screen..

[4] Brosco J.P., Seider M.I., Dunn A.C. (2006). Universal newborn screening and adverse medical outcomes: A historical note. Ment. Retard. Dev. Disabil. Res. Rev..

[5] Kanungo S., Patel D.R., Neelakantan M., Ryali B. (2018). Newborn screening and changing face of inborn errors of metabolism in the United States. Ann. Transl. Med..

[6] The government has your baby’s DNA-CNN-Com. http://www.cnn.com/2010/HEALTH/02/04/baby.dna.government/index.html.

[7] Faden R.R., Holtzman N.A., Chwalow A.J. (1982). Parental rights, child welfare, and public health: The case of PKU screening. Am. J. Public Health.

[8] Acuff K.L., Faden R.R. (1991). A history of prenatal and newborn screening programs: Lessons for the future. Analytic.

[9] Reinstein E. (2015). Challenges of using next generation sequencing in newborn screening. Genet. Res..

[10] Hollegaard M.V., Grauholm J., Nielsen R., Grove J., Mandrup S., Hougaard D.M. (2013). Archived neonatal dried blood spot samples can be used for accurate whole genome and exome-targeted next-generation sequencing. Mol. Genet. Metab..

[11] Relizani K., Goyenvalle A. (2018). The Use of Antisense Oligonucleotides for the Treatment of Duchenne Muscular Dystrophy. Methods Mol. Biol..

[12] Berg J.S., Agrawal P.B., Bailey D.B., Beggs A.H., Brenner S.E., Brower A.M., Cakici J.A., Ceyhan-Birsoy O., Chan K., Chen F. (2017). Newborn Sequencing in Genomic Medicine and Public Health. Pediatrics.

[13] Goldenberg A.J., Lloyd-Puryear M., Brosco J.P., Therrell B., Bush L., Berry S., Brower A., Bonhomme N., Bowdish B., Chrysler D. (2019). Including ELSI research questions in newborn screening pilot studies. Genet. Med..

